# THE LONG-TERM RELATIONSHIP BETWEEN SENIOR HOUSING, SOCIAL ISOLATION, AND COGNITIVE DECLINE

**DOI:** 10.1093/geroni/igae098.1962

**Published:** 2024-12-31

**Authors:** Sojung Park, Gyeahyung Jeon, Eunsun Kwon, Ji young Kang

**Affiliations:** Washington University in St. Louis, St. Louis, Missouri, United States; Hannam University, Daejeon, Republic of Korea; Washington University in St. Louis, St. Louis, Missouri, United States; Chungnam National University, Daejeon, Republic of Korea

## Abstract

Community-based senior housing has been advocated as an alternative living environment that promotes a sense of community. This study explores the long-term relationship between living in senior housing, social connection, and cognitive decline. Data came from the 12 waves (year 2010-2022) of the National Health and Aging Trends Study (NHATS) in the U.S. to compare two groups (Traditional Housing and Senior Housing) of older individuals aged 70 and over (Obs=50,128 in TH & 6078 in SH). We analyzed a long-term pattern of social isolation and cognitive function and examined the impacts of social isolation on changes in cognitive function by income groups. Weighted propensity score matching to ensure comparability between the two groups. To focus on the relations during the pandemic, we conducted a Synthetic Difference in Difference (SDID) analysis. In both housings, social isolation increased, and cognitive function decreased over a 12-year period. The regression analysis showed a clear pattern of drastic drops and recovery in the level of social isolation and cognitive function. Compared to senior housing, residents in traditional housing were likely to experience increased social isolation [coef: 0.20, p<0.001] and decreased cognitive function [coef: -0.79, p<0.01]. A series of subgroup analyses showed the protective effect of senior housing was pronounced for low-income older adults. With the long-term evidence about the protective effects of senior housing within the context of the COVID-19 pandemic, our study provides a rigorous basis for much-needed future research to identify core factors and mechanisms for healthy aging among low-income senior housing residents

